# Effect of a low-calorie diet on 24-hour urinary parameters of obese adults with idiopathic calcium oxalate kidney stones

**DOI:** 10.1590/S1677-5538.IBJU.2021.0140

**Published:** 2021-06-25

**Authors:** Alexandre Danilovic, Giovanni Scala Marchini, Nidia Denise Pucci, Brian Coimbra, Fabio Cesar Miranda Torricelli, Carlos Batagello, Fabio Carvalho Vicentini, Miguel Srougi, William C. Nahas, Eduardo Mazzucchi

**Affiliations:** 1 Faculdade de Medicina da Universidade de São Paulo Hospital das Clínicas Departamento de Urologia São PauloSP Brasil Departamento de Urologia, Hospital das Clínicas, Faculdade de Medicina da Universidade de São Paulo - FMUSP, São Paulo, SP, Brasil; 2 Faculdade de Medicina da Universidade de São Paulo Hospital das Clínicas Departamento de Nutrição São PauloSP Brasil Departamento de Nutrição, Hospital das Clínicas, Faculdade de Medicina da Universidade de São Paulo - FMUSP, São Paulo, SP, Brasil

**Keywords:** Kidney Calculi, Urolithiasis, Diet

## Abstract

**Purpose:**

to evaluate the effect of low-calorie diet on 24-hour urinary metabolic parameters of obese adults with idiopathic calcium oxalate kidney stones.

**Materials and Methods:**

Adult idiopathic calcium oxalate stone formers, with body mass index (BMI) ≥30kg/m^2^ and a known lithogenic metabolic abnormality, were submitted to low-calorie diet for twelve weeks. After enrolment, anthropometric measures, serum exams, 24-hour urinary metabolic parameters and body impedance were collected one month prior to dietary intervention and at the end of twelve weeks. Correlations between weight loss, waist circumference loss, fat loss and variation in 24-hour urinary lithogenic parameters and calcium oxalate urinary supersaturation (CaOx SS) as per Tiselius equation were analysed.

**Results:**

From January 2017 to January 2018, 39 patients were enrolled to participate in this study. Median (range) prescribed diet was 1300 (1100-2100) Kcal/day. Mean age was 51.7±11.0 (29-68) years old and 69.2% were female. 30.8% of the participants shifted from obesity to BMI <30kg/m^2^ and none to BMI <25kg/m^2^. A significant correlation was found between baseline 24-hour urinary oxalate and weight (p=0.018) and BMI (p=0.026). No correlation was found between variation of weight, waist circumference, fat mass and 24-h urinary stone risk factors or CaOx SS.

**Conclusions:**

Short-term modest weight loss induced by twelve weeks of low-calorie diet is not associated with a decrease of 24-hour urinary lithogenic parameters in idiopathic calcium oxalate stone formers. Calcium oxalate urinary stone formation is probably multifactorial and driven by other factors than weight.

## INTRODUCTION

Epidemiological evidence suggests that the increasing prevalence of kidney stone disease may be associated with the uprising prevalence of obesity. Between 1988 and 2010, the prevalence of urolithiasis in the United States of America increased from 5.2% to 8.8% whereas the prevalence of obesity increased from 22.5% to 37.4% between 1988 and 2014 (1, 2). Moreover, it has been demonstrated that urolithiasis is more common among obese than normal weight individuals (1).

Obesity is an important public health concern as it is a major contributor for many life-threatening diseases such as type II diabetes, hypertension, sleep apnea and heart disease. Weight loss is a well-established therapy to mitigate mortality and risk factors related to obesity. In obese adults, intentional weight loss may be associated with approximately 15% reduction in all-cause mortality (3). It is believed that a sustained reduction as modest as 3% to 5% of the body weight is already beneficial in order to reduce some of the risks associated to obesity (4). However, some comorbid conditions need a reduction of 10% to 15% to translate into clinical improvement (5).

Calcium oxalate is the most common composition of urinary stones in obese and in normal weight stone formers. Although more than 62% of urinary stones in obese stone formers are composed of calcium oxalate, the proportion of uric acid stones gradually increases with body mass index (BMI) (6). Increasing body mass index has been related to several urinary risk factors for kidney stone disease (7, 8). Consistent data showed that urinary pH is inversely related to BMI in stone formers being responsible for increasing proportion of uric acid stones (9). However, data showing an association between BMI and calcium oxalate stones is less consistent. Currently, standard diet recommended for idiopathic calcium oxalate stone formers is normal amount of calcium, fluid intake >2.5L and reduced intake of sodium and protein (10). There is no established correlation between weight loss and urinary changes in obese calcium oxalate stone formers (11). Our hypothesis was that weight loss could decrease urinary risk factors in obese adults with calcium oxalate kidney stones. Therefore, our main purpose was to evaluate the effect of a low-calorie diet on 24-hour urinary parameters of obese adults with calcium oxalate kidney stones and a known urinary lithogenic abnormality.

## MATERIALS AND METHODS

### Study design

#### Inclusion and Exclusion Criteria

Idiopathic calcium oxalate stone formers >18 year-old, with a body mass index (BMI) ≥30kg/m^2^ and at least one 24-hour urinary lithogenic abnormality other than low urinary volume, were accessed to join this study. Patients with psychiatric disorders, repeated urinary tract infection, stone composition other than calcium oxalate, chronic renal failure (estimated glomerular filtration rate-eGFR <60mL/min), submitted to previous surgery to treat obesity or under use of thiazide, citrate, or allopurinol were excluded from the study. The institutional ethics committee approved the study protocol (Institutional Review Board Number 13415) and written informed consent was obtained from all patients according to the Declaration of Helsinki Ethical Principles for Medical Research involving Human Subjects. This study was conducted in a dedicated urinary stone unit of a university hospital.

Previous urinary stone status was confirmed by computed tomography and a stone analysis made no more than a month prior enrolment confirmed >50% calcium oxalate in all included subjects. Abnormalities considered in at least one valid 24-hour urinary collection were calcium, oxalate, citrate and magnesium.

### Dietary Recommendations

Patients were recommended an individualized meal plan which consisted of daily ingestion of a low-calorie diet (16kcal/kg BW/day) for twelve weeks in addition to standard recommendation of normal daily intake of calcium (800mg-1200mg), fluid intake >2.5L, and reduced intake of sodium (<2.3g Na or 6g NaCl) and protein (<1.2g/Kg BW) (10, 12, 13). Individualized meal plans were created at baseline using distribution of macronutrients (55% carbohydrates, 15% proteins, 30% fat) (14). Patients were individually and personally evaluated by a registered dietitian each month. Daily food record of each patient was used to evaluate diet compliance. Anthropometric measures, serum exams, 24-hour urinary lithogenic parameters and body impedance data (InBody Co. Korea) were collected four weeks prior to dietary intervention and at the end of the twelve week study period (15-17).

### Analysed Parameters

Only 24-hour urinary samples containing urinary creatinine between 1.040 - 2.350mg/24h for men and 740 - 1.570mg/24h for women were considered valid. The standardized laboratory values used were: hypercalciuria >250mg/24h of calcium excretion for men and >200mg/24h for women; hyperoxaluria >31mg/24h oxalate excretion; hypocitraturia <320mg/24h citrate excretion; hypomagnesuria <60mg/24h magnesium excretion.

Baseline weight, BMI, waist circumference and body fat measured by impedance were correlated with baseline 24-hour urinary lithogenic parameters. Correlations between weight loss, waist circumference loss and fat loss on body impedance and variation in 24-hour urinary lithogenic parameters and calcium oxalate urinary supersaturation (CaOx SS) as per Tiselius equation were analysed (4, 18).

Tiselius equation for calcium oxalate supersaturation:

$$\text{AP}(\text{CaOx})\text{index}=1.9\text{X}{\left[\text{Ca}\right]}^{0.84}\text{X}[\text{Ox}]\text{X}{[\text{Cit}]\text{-}}^{0.22}\text{X}{\left[\text{Mg}\right]}^{-0.12}\text{X}{\left[\text{Vol}\right]}^{-1.03}$$

## STATISTICAL ANALYSIS

Categorical data were reported as frequency and percentage and continuous data as mean and standard deviation. For comparison between pre-intervention and post-intervention parameters in the same patient, a paired T-Test was used for continuous variables and a Chi-Square test was used for categorical variables. For sub analysis purposes, we used a Student T-Test when comparing continuous variables. All correlations were performed using Spearman's rho to evaluate association between overall parameters and result's variations. SPSS® Statistics Version 25 (IBM Corp©, USA) was used for statistical analysis. Significance was set at p <0.05.

## RESULTS

### Population Demographics

**Figure 1:**
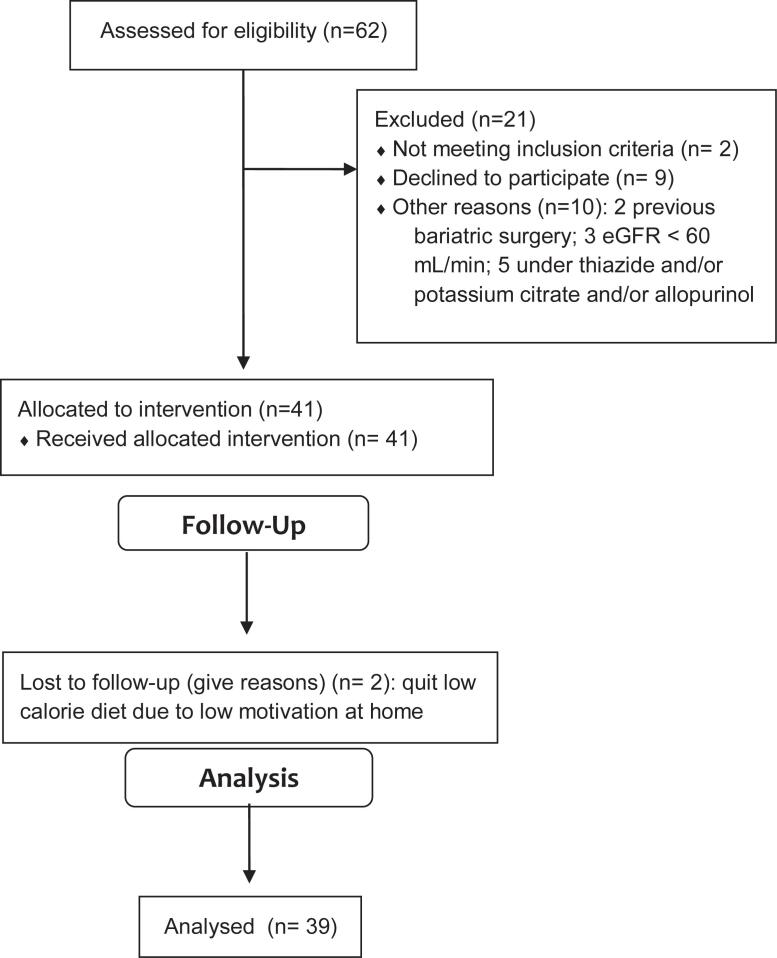
Flow diagram.

From January 2017 to January 2018, sixty-two patients were assessed for eligibility. After initial enrolment, 39 patients respected inclusion and exclusion criteria and were included in our study (Figure-1). Median (range) prescribed diet was 1300 (1100 - 2100) Kcal/day. Mean age was 51.7±11.0 (29 - 68) years old and 69.2% were female. Table-1 depicts demographic data of the studied population.

**Table 1:** Baseline characteristics of the study population.

Feature				Baseline (N=39)
Female, N (%)				27 (69.2)
Age (mean ± SD), years (range)				51.7 ± 11.0 (29-68)
BMI (mean ± SD), Kg/m^2^				34.7 ± 5.3
Abdominal circumference (mean ± SD), cm				109.4 ± 12.7
Glycosylated hemoglobin (mean ± SD), %				6.1 ± 1.1
**Glycosylated hemoglobin, N (%)**				
			5.7 - 6.5	8 (20.5)
			>6.5	8 (20.5)
			>7.0	7 (17.9)
**Hypertension, mmHg N (%)**				
		SBP ≥ 140 and/or DBP ≥ 90		7 (17.9)
LDL ≥ 160 mg/dL, N (%)				3 (7.7)
HDL < 40 mg/dL Male, < 50 mg/dL Female, N (%)				19 (48.7)
Triglycerides ≥ 150 mg/dL, N (%)				19 (48.7)
eGFR, mean ± SD (Female/Male), mL/min				107.1 ± 33.4/ 126.0± 39.3
Fat mass (mean ± SD), %				42.8 ± 6.7
**Stone composition**				
	Calcium oxalate monohydrate, N (%)			22 (56.4)
	Calcium oxalate dihydrate, N (%)			17 (43.6)
**Urinary lithogenic abnormality**				
	Low volume, N (%)			35 (90)
	Hypercalciuria, N (%)			17 (44)
	Hyperoxaluria, N (%)			11 (28)
	Hypocitraturia, N (%)			23 (59)

### Baseline Analysis

Calcium oxalate monohydrate and calcium oxalate dihydrate were the stone composition of 56.4% and 43.6% of the participants, respectively. Hypercalciuria, Hyperoxaluria and Hypocitraturia were found in 44%, 28% and 59% of patients, respectively (Table-1). Spearman's correlations between baseline 24-hour urinary lithogenic parameters and baseline weight, BMI, waist circumference and body fat demonstrated a significant correlation between 24-hour urinary oxalate and weight (p=0.018) and BMI (p=0.026).

### Overall Interventional Outcomes

There was a significant reduction on mean weight (p <0.001), BMI (p <0.001), waist circumference (p <0.001), body fat mass (p <0.001) and CaOx SS (p=0.021) (Table-2). No significant variation was noticed for other parameters. After twelve weeks, 30.8% of the participants shifted from obesity to BMI <30kg/m^2^ and none to BMI <25kg/m^2^. In total, 53.8% and 38.5% of the patients achieved >3% and >5% weight loss after 12 weeks of low-calorie diet.

**Table 2:** Metabolic features of pre vs. post-low calorie diet.

Features	Pre-diet (N=39)	Post-diet (N=39)	p
Weight (mean ± SD), Kg	89.2 ± 16.2	85.7± 16.7	<0.001
BMI (mean ± SD), Kg/m^2^	34.7±5.3	33.3 ± 5.4	<0.001
Waist circumference (mean ± SD), cm	109.4 ± 12.7	104.5 ± 12.2	<0.001
Glycosylated hemoglobina (mean ± SD), %	6.1± 1.1	5.9 ± 0.9	0.183
**Total cholesterol (mean ± SD), mg/dL**	**189.3± 42.5**	**191.2 ± 47.4**	**0.703**
HDL (mean ± SD), mg/dL	47.5± 13.8	48.6 ± 13.0	0.343
LDL (mean ± SD), mg/dL	111.4 ± 38.0	115.1 ± 44.2	0.449
Triglycerides (mean ± SD), mg/dL	158.6 ± 87.8	152.1 ± 104.6	0.509
Uric acid (mean ± SD), mg/dL	5.6 ± 1.5	5.3 ± 1.2	0.068
Creatinine (mean ± SD), mg/dL	0.9 ± 0.2	0.9 ± 0.3	0.849
Venous pH (mean ± SD)	7.37 ± 0.04	7.36 ± 0.03	0.107
**Total calcium (mean ± SD), mg/dL**	**9.5 ± 0.4**	**9.6 ± 0.4**	**0.199**
PTH (mean ± SD), pg/mL	48.6 ± 19.7	49.4 ± 24.1	0.700
Cholecalciferol (mean ± SD), nmol/L	23.6 ± 8.9	23.5 ± 7.4	0.981
Urinary volume (mean ± SD), mL	1559.0 ± 440.1	1771.0 ± 501.1	0.007
Urinary density (mean ± SD)	1017.4 ± 5.4	1017.6 ± 5.8	0.918
Urinary pH (mean ± SD)	5.5 ± 0.7	5.7 ± 0.8	0.242
Urinary calcium (mean ± SD), mg/day	218.5 ± 134.1	216.0 ± 128.8	0.880
Urinary oxalate (mean ± SD), mg/day	24.8 ± 11.8	23.3 ± 12.3	0.483
Urinary citrate (mean ± SD), mg/day	362.4 ± 305.3	449.8 ± 224.4	0.056
Urinary magnesium (mean ± SD), mg/day	80.5 ± 34.9	83.6 ± 39.0	0.619
Calcium oxalate SS (mean ± SD)	1.2 ± 1.0	0.9 ± 0.7	0.021
Fat mass (mean ± SD), %	42.4 ± 6.7	40.8 ± 7.3	<0.001

A significant correlation was found between urinary pH variation and waist circumference variation (R=-0.330; p=0.043). No significant correlation was found between weight loss and variation of 24-hour urinary calcium (p=0.072), oxalate (p=0.080), citrate (p=0.206), magnesium (p=0.356) and CaOxSS (p=0.266). No correlation was found between variation of waist circumference and variation of 24-hour urinary calcium (p=0.160), oxalate (p=0.600), citrate (p=0.651), magnesium (p=0.718) and CaOxSS (p=0.154). No correlation was found between body fat loss and variation of 24-hour urinary calcium (p=0.712), oxalate (p=0.873), citrate (p=0.409), magnesium (p=0.087) and CaOxSS (p=0.609) (Table-3).

**Table 3:** Spearman's correlation between change of obesity parameters and change of calcium oxalate urinary stone risk factors.

Δ 24 hour urinary parameter	Δ Waist circumference (p value)	Δ Weight (p value)	Δ Fat (p value)
Volume	-0.068 (0.684)	-0.007 (0.968)	-0.066 (0.725)
pH	-0.330 (0.043)	0.118 (0.475)	0.306 (0.094)
Sodium	0.141 (0.398)	-0.205 (0.210)	-0.264 (0.152)
Calcium	0.233 (0.160)	0.292 (0.072)	0.069 (0.712)
Oxalate	-0.088 (0.600)	-0.284 (0.080)	0.030 (0.873)
Citrate	-0.076 (0.651)	-0.207 (0.206)	-0.154 (0.409)
Magnesium	-0.060 (0.718)	0.152 (0.356)	0.312 (0.087)
Uric acid	-0.085 (0.611)	0.031 (0.853)	-0.265 (0.150)
CaOxSS	0.239 (0.154)	-0.185 (0.266)	-0.097 (0.609)

Sub analysis of stone composition: calcium oxalate dihydrate and calcium oxalate monohydrate

There were 17 and 22 participants in our study with stones composed of calcium oxalate dehydrate and calcium oxalate monohydrate, respectively. Variations of 24-hour urinary lithogenic parameters after low calorie diet were compared between groups. Only variation of urinary excretion of magnesium was different between groups. Patients with calcium oxalate dihydrate stones increased whereas patients with calcium oxalate monohydrate stones decreased the 24-hour urinary excretion of magnesium (19.6±45.0 vs. -9.6±28.5mg/day, p=0.037, respectively) (supplementary Table-1). However, a sub analysis within these groups could not find a correlation between change of 24-hour urinary parameters and loss of weight, waist circumference or fat mass (supplementary Tables 2 and 3).

**Supplementary Table 1:** Comparison of variations of 24-hour urinary lithogenic parameters after low calorie diet between groups of calcium oxalate stone composition.

Δ Features	Calcium oxalate dihydrate (N=17)	Calcium oxalate monohydrate (N=22)	p
Weight (mean ± SD), Kg	- 4.2 ± 3.1	- 3.0 ± 2.2	0.120
%BMI (mean ± SD), Kg/m^2^	- 4.9 ± 3.5	- 3.5 ± 2.7	0.123
Waist circumference (mean ± SD), cm	- 5.1 ± 5.7	- 4.5 ± 7.1	0.824
Urinary volume (mean ± SD), mL	265.3 ± 409.3	171.6 ± 505.5	0.730
Urinary pH (mean ± SD)	0.2 ± 1.1	0.1 ± 0.9	0.776
Urinary calcium (mean ± SD), mg/day	27.8 ± 94.2	- 26.0 ± 110.0	0.120
Urinary oxalate (mean ± SD), mg/day	- 2.1 ± 15.9	- 1.1 ± 11.4	0.517
Urinary citrate (mean ± SD), mg/day	173.1 ± 224.0	21.0 ± 298.6	0.119
Urinary magnesium (mean ± SD), mg/day	19.6 ± 45.0	-9.6 ± 28.5	0.037
Calcium oxalate SS (mean ± SD)	- 0.3 ± 0.6	- 0.2 ± 0.8	0.621
Fat mass (mean ± SD), %	- 7.9 ± 7.0	- 6.1 ± 6.0	0.386

**Supplementary Table 2:** Correlation between 24-hour urinary parameters change and weight change, abdominal circumference change and fat mass change in 17 patients with calcium oxalate dihydrated stones.

Δ 24 hour urinary parameter	Δ Waist circumference (p value)	Δ Weight (p value)	Δ Fat (p value)
Volume	-0.237 (0.376)	-0.053 (0.841)	-0.308 (0.330)
pH	-0.363 (0.166)	-0.154 (0.555)	0.084 (0.796)
Sodium	0.043 (0.875)	-0.147 (0.573)	-0.448 (0.145)
Calcium	0.161 (0.553)	0.206 (0.428)	-0.161 (0.617)
Oxalate	-0.137 (0.614)	-0.375 (0.138)	-0.168 (0.601)
Citrate	0.152 (0.573)	-0.392 (0.119)	-0.573 (0.051)
Magnesium	-0.266 (0.319)	0.181 (0.488)	0.389 (0.212)
Uric acid	-0.140 (0.606)	-0.253 (0.328)	-0.329 (0.296)
CaOxSS	0.155 (0.566)	-0.414 (0.098)	-0.322 (0.308)

**Supplementary Table 3:** Correlation between 24-hour urinary parameters change and weight change, abdominal circumference change and fat mass change in 22 patients with calcium oxalate monohydrate stones.

Δ 24 hour urinary parameter	Δ Waist circumference (p value)	Δ Weight (p value)	Δ Fat (p value)
Volume	0.037 (0.869)	-0.054 (0.813)	0.205 (0.399)
pH	-0.385 (0.077)	0.391 (0.072)	0.427 (0.068)
Sodium	0.188 (0.401)	-0.268 (0.227)	-0.197 (0.420)
Calcium	0.205 (0.361)	0.475 (0.026)	0.246 (0.311)
Oxalate	-0.010 (0.966)	-0.407 (0.060)	0.025 (0.920)
Citrate	-0.353 (0.107)	0.022 (0.923)	0.033 (0.892)
Magnesium	-0.102 (0.652)	0.255 (0.252)	0.408 (0.083)
Uric acid	-0.078 (0.730)	0.252 (0.258)	-0.137 (0.576)
CaOxSS	0.332 (0.142)	0.006 (0.978)	0.022 (0.932)

### Sub analysis within genders

There were 27 females and 12 male participants in our study. Variations of 24-hour urinary lithogenic parameters after low calorie diet did not differ between groups (supplementary Table-4). A sub analysis within these groups could not find a correlation between change of 24-hour urinary parameters and loss of weight, waist circumference or fat mass (supplementary Tables 5 and 6).

**Supplementary Table 4:** Comparison of variations of 24-hour urinary lithogenic parameters after low calorie diet between genders.

Δ Features	Female (N=27)	Male (N=12)	p
Weight (mean ± SD), Kg	- 3.5 ± 2.6	- 3.4 ± 2.9	0.889
%BMI (mean ± SD), Kg/m^2^	- 4.3 ± 3.1	- 3.6 ± 3.2	0.505
Waist circumference (mean ± SD), cm	- 5.2 ± 7.0	- 3.6 ± 4.8	0.437
Urinary volume (mean ± SD), mL	139.8 ± 411.1	385.8 ± 545.4	0.198
Urinary pH (mean ± SD)	0.1 ± 1.0	0.4 ± 0.7	0.226
Urinary calcium (mean ± SD), mg/day	3.0 ± 100.6	- 15.0 ± 119.9	0.656
Urinary oxalate (mean ± SD), mg/day	- 2.1 ± 15.7	- 0.3 ± 5.8	0.600
Urinary citrate (mean ± SD), mg/day	68.2 ± 280.4	130.4 ± 273.0	0.522
Urinary magnesium (mean ± SD), mg/day	3.0 ± 43.4	3.4 ± 28.3	0.972
Calcium oxalate SS (mean ± SD)	- 0.2 ± 0.7	- 0.4 ± 0.7	0.373
Fat mass (mean ± SD), %	- 6.3 ± 5.0	- 7.9 ± 9.1	0.632

**Supplementary Table 5:** Correlation between 24-hour urinary parameters change and weight change, abdominal circumference change and fat mass change in 27 females.

Δ 24 hour urinary parameter	Δ Waist circumference (p value)	Δ Weight (p value)	Δ Fat (p value)
Volume	-0.203 (0.311)	0.172 (0.391)	0.123 (0.585)
pH	-0.030 (0.883)	-0.088 (0.661)	0.311 (0.158)
Sodium	0.146 (0.467)	-0.170 (0.398)	-0.457 (0.033)
Calcium	0.254 (0.202)	0.222 (0.265)	-0.156 (0.489)
Oxalate	0.019 (0.924)	-0.113 (0.575)	0.083 (0.714)
Citrate	0.136 (0.499)	-0.359 (0.066)	-0.362 (0.098)
Magnesium	-0.001 (0.996)	-0.057 (0.776)	0.001 (0.997)
Uric acid	0.142 (0.480)	-0.183 (0.258)	-0.533 (0.011)
CaOxSS	0.156 (0.446)	-0.070 (0.735)	-0.043 (0.853)

**Supplementary Table 6:** Correlation between 24-hour urinary parameters change and weight change, abdominal circumference change and fat mass change in 12 males.

Δ 24 hour urinary parameter	Δ Waist circumference (p value)	Δ Weight (p value)	Δ Fat (p value)
Volume	0.201 (0.553)	-0.301 (0.342)	-0.317 (0.406)
pH	-0.483 (0.132)	0.416 (0.179)	0.639 (0.064)
Sodium	0.030 (0.931)	-0.413 (0.182)	-0.075 (0.847)
Calcium	0.172 (0.613)	0.105 (0.745)	0.293 (0.444)
Oxalate	-0.377 (0.253)	-0.439 (0.154)	-0.084 (0.831)
Citrate	-0.435 (0.181)	0.042 (0.897)	0.350 (0.356)
Magnesium	-0.166 (0.625)	0.092 (0.777)	0.494 (0.177)
Uric acid	-0.560 (0.073)	0.049 (0.880)	0.527 (0.145)
CaOxSS	-0.151 (0.658)	-0.140 (0.665)	0.100 (0.798)

## DISCUSSION

Short-term modest weight loss induced by a twelve weeks of low-calorie diet in addition to standard recommended diet for stone patients is not associated with a decrease of 24-hour urinary lithogenic parameters in obese adults with idiopathic calcium oxalate kidney stones. No correlation was found between weight loss, waist circumference change, fat loss and 24-hour urinary calcium, oxalate, citrate, magnesium or calcium oxalate supersaturation.

Previous studies have demonstrated that urolithiasis is more common among obese than normal weight individuals and that there is a correlation between increasing BMI and increasing stone risk factors (1, 7, 8, 19). Actually, most cited studies are cross-sectional in their methodology and preclude for a cause-effect conclusion. Association is different than causality and only prospective interventional studies allow for such inference. Calcium oxalate is the most prevalent stone composition regardless of BMI and is the main composition of 54.4% to 71.5% of the obese stone formers (6, 20). However, the proportion of uric acid composition gradually increases with BMI (6). Maalouf et al. demonstrated that urinary pH is inversely related to weight. Lower urinary pH due to insulin resistance of obesity explains the increased proportion of uric acid stones in obese stone formers (9). We found that urinary pH may be inversely related to waist circumference in obese idiopathic calcium oxalate stone formers but we could not found a correlation between variation of urinary pH and weight loss or fat loss. Although calcium oxalate is the most prevalent stone composition among obese patients, the association between idiopathic calcium stone formers and obesity is less consistent. DASH-style diet was associated with reduced risk for kidney stones (21). But no study to date proved that decreasing BMI could decrease kidney stone formation. Torricelli et al. have demonstrated that dietary recommendations directed to stone prevention are equally effective in obese and non-obese kidney stone formers (22). Therefore, the objective of this study was to identify a correlation between weight loss, waist circumference change and fat loss with calcium oxalate urinary risk factors.

Despite other authors had found correlation between increasing BMI and increasing in 24-hours urinary stone risk factors, this study could not demonstrate that decreasing weight is associated to a reduction in 24-hour urinary risk factors in obese idiopathic calcium oxalate stone formers (7, 8). We studied obese idiopathic calcium oxalate stone formers with at least one known abnormality in 24-hour urinary stone risk factors without current medical treatment. Hypercalciuria was present in 44% of the participants, hyperoxaluria in 28% and hypocitraturia in 59%. Calcium oxalate monohydrate and calcium oxalate dihydrate were the stone composition of 56.4% and 43.6% of the participants, respectively. Previous studies from other authors demonstrated that patients with greater BMIs excrete more urinary oxalate, uric acid, sodium and phosphate than participants with lower BMIs and has an inverse relation with urine pH. However, no correlation between BMI and urinary supersaturation of calcium oxalate was found. The authors concluded that the greater incidence of kidney stones in the obese may be due to an increase in uric acid nephrolithiasis and not calcium oxalate stones (23). Likewise, we found an association between weight and BMI and 24-hour urinary oxalate that may be explained in part by the higher proportion of stones composed of calcium oxalate monohydrate in our studied population. Other baseline associations with urinary pH, calcium, citrate, magnesium or calcium oxalate supersaturation were not significant.

The absence of correlation between weight loss and 24-hours urinary parameters of calcium oxalate stone formers suggests that factors other than obesity are implicated in the physiology of calcium oxalate stone formation. Afkari et al. suggest that probiotic bacteria may restore gut microbiota balance and reduce urinary oxalate excretion (24). Our study demonstrated that weight is associated with urinary oxalate excretion. Therefore, obese patients with calcium oxalate stones may benefit from strategies aiming to reduce urinary oxalate excretion. Moreover, regional differences should be observed. Obesity is not a stone risk factor for every population. The rates of obesity and overweight in renal stone formers in Italy are similar to rates reported in the general population (25).

This study has limitations. This prospective study has no control treatment or randomization process and the number of participants is low. However, it is very difficult to enrol obese patients with idiopathic calcium oxalate kidney stones without previous medical treatment willing to accept the challenge of a low-calorie diet. The aim of this study was to identify a correlation between weight loss and abnormalities in 24-hour urinary parameters. We were not willing to study the efficacy of the low-calorie diet. It was only the method to induce weight reduction. After twelve weeks, only 30.8% of the participants shifted from obesity to BMI <30kg/m^2^ and none to BMI <25kg/m^2^. Also, 53.8% and 38.5% of the patients achieved >3% and >5% weight loss after 12 weeks of low-calorie diet, respectively. These figures may seem low but real-world clinical practice demonstrates anti-obesity medications are associated with clinically meaningful weight loss of 2% to 4% after 12 weeks (26), highlighting the difficulty of weight loss without bariatric surgery. The final message of this study is that the correlation between weight loss and urinary lithogenic parameters of obese adults with idiopathic calcium oxalate kidney stones is still to be proven, challenging the cause-effect nature of this association. Prospective multicentre studies should be carried out to evaluate if weight gain really enhances the urinary lithogenic factors, and on the contrary, if weight loss could protect against calcium oxalate urinary stone disease.

## CONCLUSIONS

Short-term modest weight loss induced by twelve weeks of low-calorie diet is not associated with a decrease of 24-hour urinary lithogenic parameters in obese adults with idiopathic calcium oxalate kidney stone. Calcium oxalate urinary stone formation is probably multifactorial and driven by other factors than weight.

## ABBREVIATIONS USED

N: number
SD: standard deviation
Kg: kilogram
M: meter
mmHg: millimeter of mercury
SBP: systolic blood pressure
DBP: diastolic blood pressure
LDL: low-density lipoprotein
HDL: high density lipoprotein
eGFR: estimated glomerular filtration rate
mL: millimeter
min: minute
mg: milligram
dL: deciliter
nmol: nanomol
